# Supplementary figures and images for: The MAPK/ERK channel capacity exceeds 6 bit/hour
Source: PLoS Comput Biol. 2023 May 22;19(5):e1011155. doi: 10.1371/journal.pcbi.1011155 (PMC10237675; doi:10.1371/journal.pcbi.1011155)

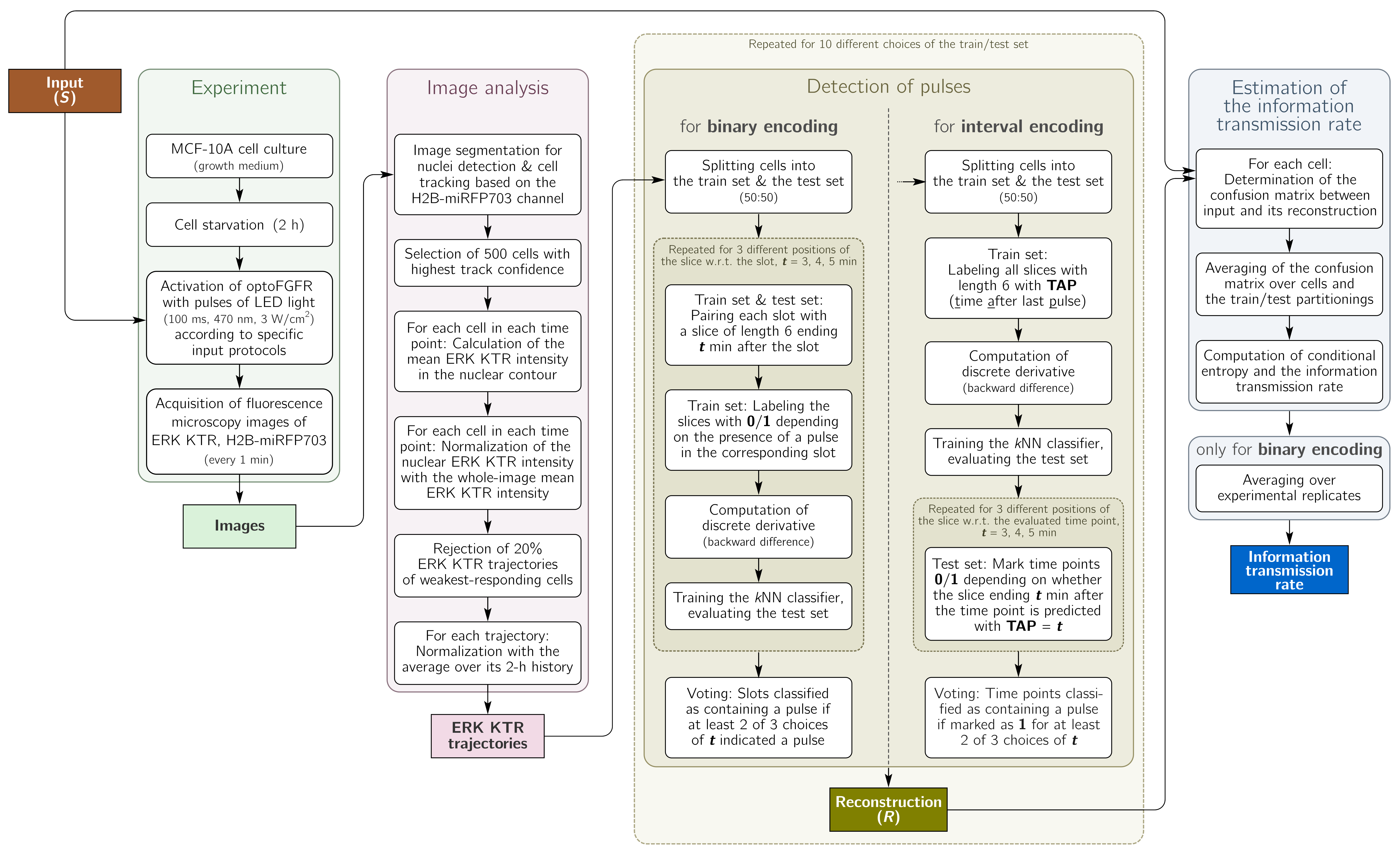

Supplement: S1 Fig — (TIF) [file pcbi.1011155.s001.tif]

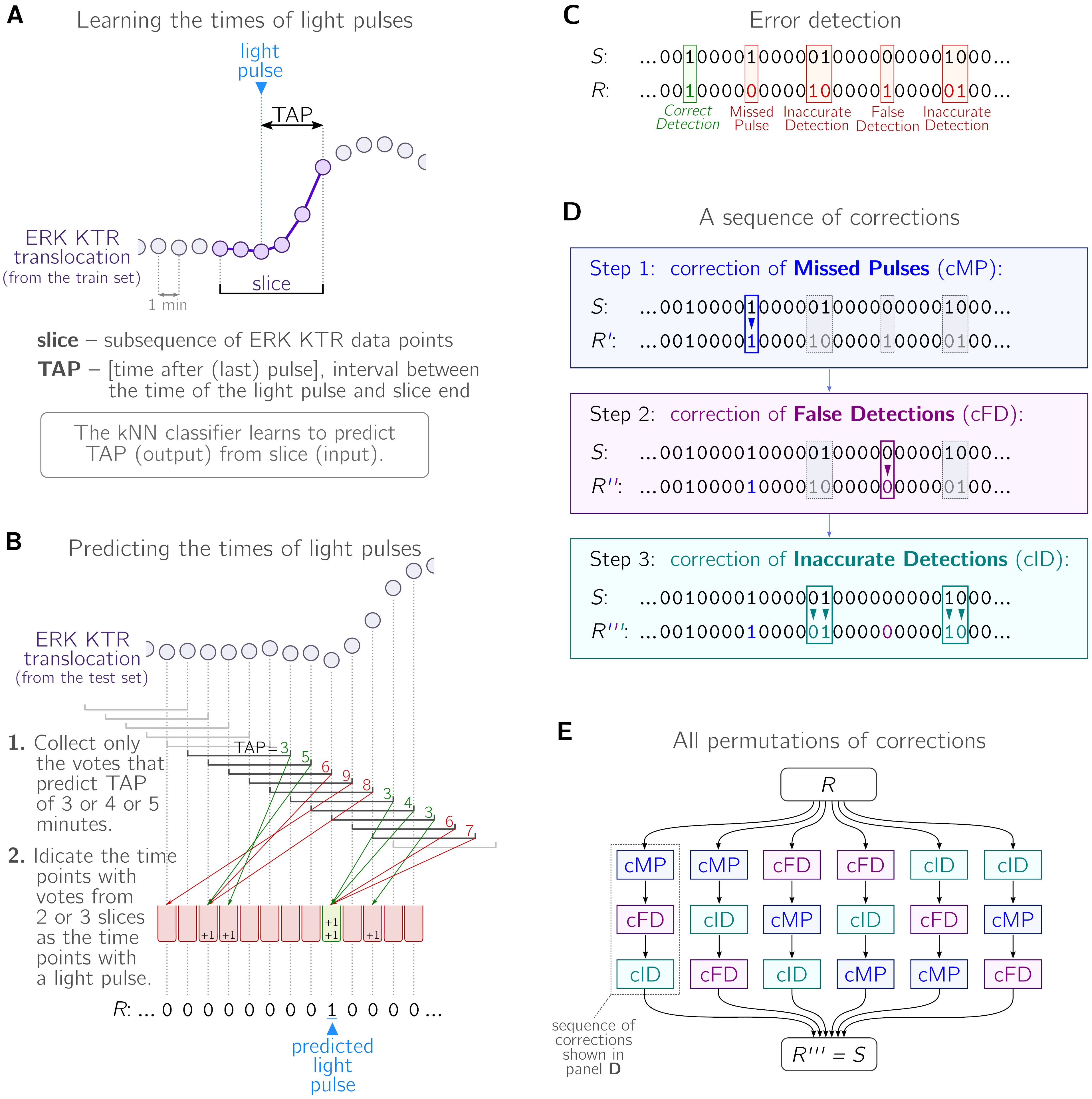

Supplement: S2 Fig — (A) Tracks from the training set are segmented into overlapping slices of 6 consecutive time points such that each time point belongs to 6 slices. Each slice is labeled with the time after pulse (TAP), measured with respect to the last time point in the slice. The kNN classifier is trained to predict the TAP based on 5 discrete differences between 6 consecutive time points of a slice. (B) For each slice, the TAP predicted by the classifier indicates a time point at which the stimulation pulse could occur. Votes for particular time points from different slices are counted. Only votes from slices predicted with TAP = 3,4,5 min are taken into account, the remaining are ignored as unreliable. Time points that received at least two out of the three possible votes are considered as time points with pulse in the final reconstruction, R. (C) Detection and labeling of errors in the reconstruction R. Inaccurate detections (one minute before or after the pulse) are not decomposed into missed pulses and false detections but assigned to their specific error types. Since the patterns ‘11’ and ‘101’ are guaranteed never to occur in the input sequence S, the error classification is unambiguous. (D) An example 3-step sequence of error corrections for the interval encoding protocols. After the third step, the fully corrected reconstruction R‴ is identical to the input sequence S. The difference between bitrate before and after each correction step is attributed to the particular information loss source. (E) As the difference in bitrate depends on the sequence of corrections, the contributions of the three types of errors are averaged over all permutations of correction steps. (TIF) [file pcbi.1011155.s002.tif]

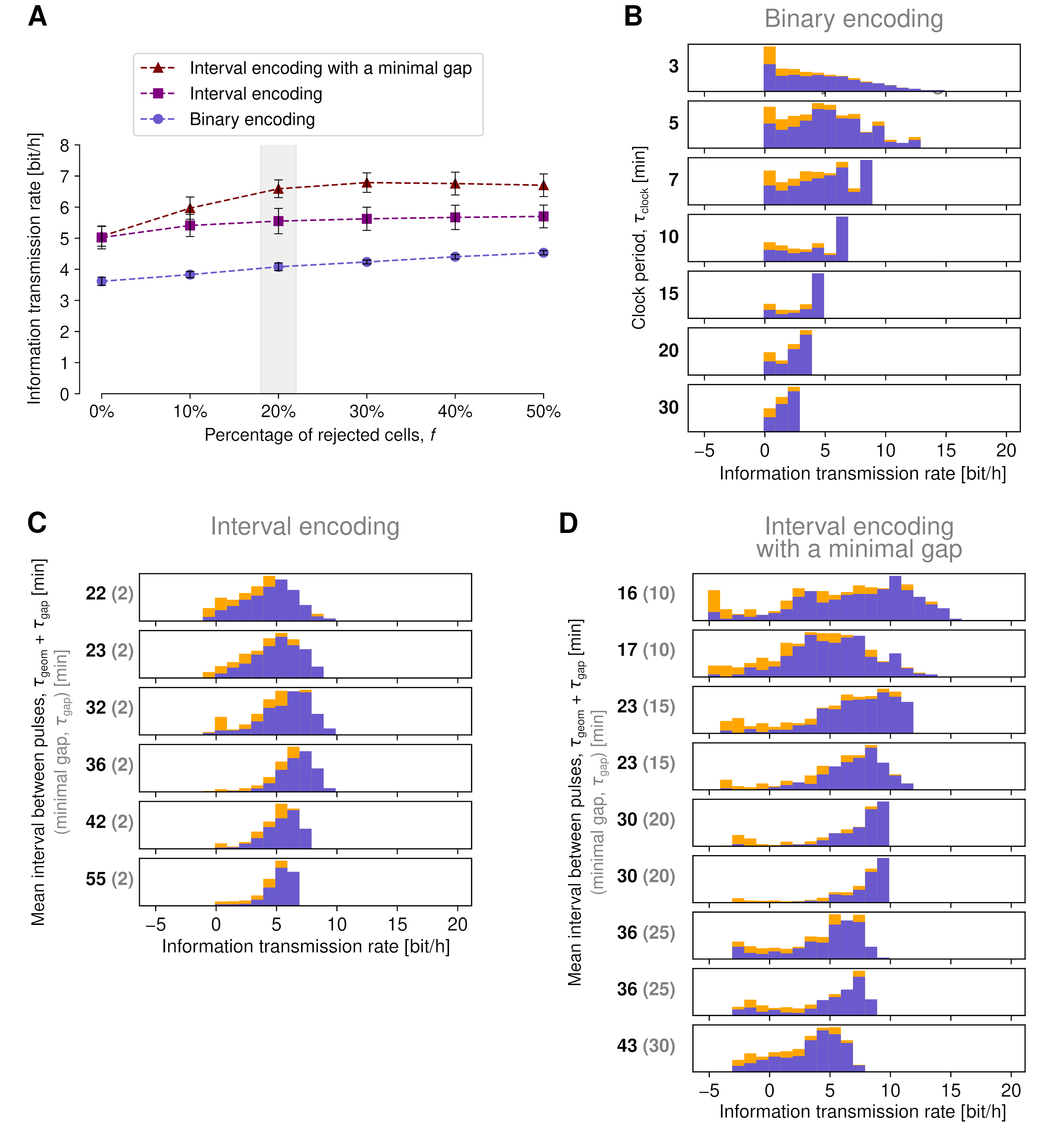

Supplement: S3 Fig — (A) Bitrate estimate as a function of the fraction of cells excluded in the preselection step. The (lower bound for) bitrate is computed as in Fig 3. Error bars denote standard error of the mean based on the 2–4 experiments with highest bitrate. In the preselection step we aim at excluding cells that do not respond to stimulation, possibly due to low expression of optoFGFR or ERK KTR. The rejection criterion is formulated in a way a priori independent of the accuracy of pulse detection, see Methods for details. Throughout the paper, the fraction of rejected cells is set to 20% (highlighted in gray), because above this value the bitrate estimates in the interval encoding protocols (with and without minimal gap) reach a plateau. (B, C, D) Histograms of the information transmission rates in single cells for (B) binary encoding, (C) interval encoding, and (D) interval encoding with a minimal gap. Estimates for cells rejected in the preselection step (20% of all cells) are marked in orange. Negative bitrate estimates can occur due to the rough approximation based on inequalities in Eq (7) in the main text. (TIF) [file pcbi.1011155.s003.tif]
