# Supplementary material for: The MAPK/ERK channel capacity exceeds 6 bit/hour
Source: PLoS Comput Biol. 2023 May 22;19(5):e1011155. doi: 10.1371/journal.pcbi.1011155 (PMC10237675; doi:10.1371/journal.pcbi.1011155)
Supplement: Attachment — Submitted filename: Nalecz-Jawecki_et_al__Response_to_Reviewers.pdf [file pcbi.1011155.s004.pdf]

Institute of Fundamental Technological Research
of the Polish Academy of Sciences
Pawińskiego 5B, 02-106 Warsaw, Poland

Department of Biosystems and Soft Matter
Laboratory of Modeling in Biology and Medicine
https://pmbm.ippt.pan.pl

Subject: **Resubmission of revised manuscript PCOMPBIOL-D-23-00079** April 5, 2023

Dear Editor, Dear Reviewers,

we appreciate your thorough reading of our manuscript and feedback. We have addressed your comments by revising our manuscript. All changes in the manuscript are highlighted in color in a separate Word file. A detailed point-by-point response to all your comments is provided below in this document. Please note that all data and computational code underlying our findings are fully available in public repositories (which was overlooked by one of the Reviewers).

In the revised manuscript, as suggested by Referee 1, we performed a reconstruction-free estimation of the bitrate to arrive at a value of about 8 bit/h (for an optimal protocol), which is about 2 bit/h higher than the estimate (from below) obtained using the reconstruction-based method. The difference is unsurprising, because unavoidably the reconstruction contains less information than the measured output. In article title we still prefer to keep the "safer" value of 6 bit/h, because it can be questioned how a cell can utilize information without performing by itself some input reconstruction.

The main question of Referee 2 is "the biological meaning of this information rate". We think that the question about the MAPK channel capacity should be considered similarly to the question of the neuron channel capacity (which in a seminal study for fly visual neuron was estimated as 90 bit/s, Ref. 14 = Strong *et al.*, 1998, *Phys. Rev. Lett.*, DOI: 10.1103/PhysRevLett.80.197). Obviously, knowing that the MAPK pathway can transmit at least 6 bit/h, one can ask how this information can be utilized. As we discussed, possibly the most information-consuming process coordinated by the MAPK pathway (mostly at the RAS level) is cell motion, however investigation of this goes beyond the scope of our study.

We hope that you will find the revised manuscript suitable for publication in *PLOS Computational Biology.*

Sincerely,
Tomasz Lipniacki
Professor and Head

Response to the Report from Reviewer 1

> This study focuses on the information transmission of ERK pathway in face of a sequence of pulses. The authors used different sequences of light pulses to activate the optoFGFR, and then measured the ERK activity through the fluorescent ERK KTR. Based on the time courses of ERK KTR translocation, authors calculated the transmitted information rate using classifier-based estimation and obtained the value of transmitted information 6 bit/hour. The experiment design and information estimation are air-tight. Aside from the tools, the main conclusion is, to me, very impressive. The 6 bit/hour is different from the transmitted information when the external stimulus is just varied once, which is usually near 1 bit. The conclusion provides insight into how the ERK pathway transfers information.

Response: We are glad to receive a favorable assessment of our study.

> While this is a thoughtful study, it can still be improved by addressing the accuracy and robustness of main conclusion. My comments are as follows:
>
> **Major comments**
>
> **1.** The authors used the output-based reconstruction to estimate the conditional entropy. However, the reconstruction seems not necessary for the estimation, because the conditional entropy can be directly estimated from the original output by using other methods, such as k-nearest neighbor (kNN) based estimators and kernel-based methods. Thus, justifications are needed to explain why the reconstruction is required for the conditional entropy estimation. Furthermore, it's unclear whether the reconstruction-based method provides an accurate estimation of conditional entropy. Therefore, a direct comparison with other widely used methods will provide a much more vigorous justification.

Response: The reconstruction is indeed dispensable for bitrate estimation. We prefer the reconstruction-based approach, in which the cell response is analyzed with a classifier, because classification results may be intelligibly interpreted as the cell capability of perceiving and reacting upon input cues. An advantage of the reconstruction-based approach is that it enables discerning particular sources of classification errors (as shown in Fig. 2B, 2D, 2F), and provides insight into information available to a responding cell. On the other hand, any classifier loses some information about responding cells and thus provides a lower bound on the estimate of the cell capability of transmitting information.

Nevertheless, motivated by the comment from Reviewer 1, we performed a direct (reconstruction-free) estimation of the information transmission rate. We used the k-nearest neighbor approach to estimate the conditional entropy $H(S|R)$ and thus $I(S, R)$. In this direct approach, the bitrate estimates are

about 2 bit/h higher than in the case of the reconstruction-based approach (*cf.* new Fig. 4 and Fig. 3). This is due to the fact that the reconstruction process itself introduces information loss, whereas in the direct approach all available information is utilized. It is debatable, however, whether cells can utilize the input information without "reconstructing the signal". Ultimately, in the revised manuscript we report the lower bound estimate obtained using both the reconstruction-based approach and the direct approach. In the direct approach we used $k = 20$, so for consistency we recalculated the reconstruction-based estimates using a kNN classifier with the same value of $k = 20$ (as a consequence, estimates shown in revised Fig. 2 and Fig. 3 marginally differ from those shown in original Fig. 2 and Fig. 3).

> 2. It is unclear how robust the main conclusion is. Therefore, error bars showing the variance of mutual information are needed, for example, in Fig 2B, 2D, 2F, and Fig 3. This will provide the robustness of the estimated mutual information value 6 bit/hour.

Response: The estimation of the information transmission rate for a single experiment is quite robust. To verify this, for Fig. 3B and 3C we performed the estimation procedure 10 times, each time differently splitting the cell trajectories into test and train data sets. For the direct estimation in Fig. 4A and 4B, which does not require a train set, we 10 times randomly selected 200 out of the 400 cell trajectories. In the revised figures, we added error bars presenting the standard deviation over those repetitions (note that sometimes the error bar is smaller than the data point marker). In Fig. 3A, where each data point is a mean of several experiments, we show standard deviation of these replicates. The main goal of the experiments was to estimate the maximum bitrate for a given type of protocol. To this end, we probed across different time intervals and the final estimate is the average over several experiments for each protocol with the highest values (encircled in Fig. 3A, 3B, 3C and new Fig. 4A, 4B). For these final estimates we give the standard error of the mean.

> 3. The authors need to go further to convey the motivation for choice of protocols. It would be helpful to describe what type of stimulus the cell usually faces, a constant stimulus or a serial of random pulse? What is the reason to consider binary encoding, interval encoding, and interval encoding with minimal gap? The authors need to justify the biological implications and choice of the three types of pulsatile stimulation protocols.

Response: Pulsatile stimulation appears to be the most generic growth-factor stimulation mode. *In vitro*, Gagliardi *et al.* [2022, *Dev. Cell*, DOI: 10.1016/j.devcel.2021.05.007] demonstrated that ERK pulses triggered locally by growth factors shed by apoptotic cells coordinate the maintenance of epithelial integrity. *In vivo* studies showed pulsatile ERK dynamics during, for example, wound healing in mice, wherein the wound edge initiates recurrent, quasiperiodic mechanochemical traveling fronts of ERK activity [Hiratsuka *et al.*, 2015, *eLife*, DOI: 10.7554/elife.05178]; during mammalian epidermis development [Hiratsuka

et al., 2020, *PNAS*, DOI: 10.1073/pnas.2006965117]; or during scale regeneration in zebrafish [De Simone *et al.*, 2021, *Nature*, DOI: 10.1038/s41586-020-03085-8].

In light of these findings, in our study we focus solely on pulsatile stimulation protocols. As we want to find an estimate of the channel capacity, we searched for a protocol that would maximize the information transmission rate. As we conjecture that protocols ensuring highest information transfer are used for cell signaling, we searched for a protocol that would maximize the information transmission rate, and thus estimated the MAPK/ERK channel capacity. We considered three different families of pulsatile input protocols: (*i*) binary encoding, resembling digital communication with zeros and ones occurring in well-defined, temporally equidistant time slots, is plausibly the simplest pulsatile stimulation protocol; (*ii*) interval encoding, with exponentially-distributed waiting times in discrete time domain (so, effectively, geometrically-distributed waiting times), is statistically maximally random (entropic); this protocol is also natural from the biological perspective as it implies constant propensity of receiving stimulation pulses in time; (*iii*) interval encoding with an additional gap after each pulse is an attempt to tailor a maximally entropic stimulation to cells characterized by an inextricable refractory (resting) period. In the case of ERK activity waves one can expect that the first "layer" of cells filters out pulses that occur within the refractory time, and thus the farther "layers" of cells will be subjected to stimulation that resembles the interval encoding with a minimal gap. We expanded Introduction and Discussion to include the above information.

> 4. It is confusing about the difference between the three protocols mentioned in Fig. 1A. The study uses geometry distribution in the second and third protocols. Since the geometry distribution with mean τ_{geom} is the probability distribution of the number X of Bernoulli trials needed to get one success, it means that a binomial trial is made at every minute and the probability of success is $1/\tau_{\text{geom}}$. Besides, the first protocol is that a binomial trial is made every 5 minutes and the probability of success is 1/2. So, if I understand correctly, the second protocol is very similar to the first protocol if τ is 2, and the third protocol is the same as the first protocol if τ_{geom} is 2 and τ_{gap} is 5.

Response: In the second family of protocols (interval encoding), the geometric probability distribution of inter-pulse intervals may be equivalently expressed in terms of Bernoulli trials (made every minute), whereas in the third family of protocols (interval encoding with a gap) the Bernoulli/binomial trials are prohibited for a defined time span after a successful trial (input pulse). The difference between the first and the second family of protocols is such that in the first family, Bernoulli trails are made every τ_{clock} (constant for an experiment and varied between experiments) with constant probability 1/2, while in the second family of protocols, trials are made every minute with probality $1/\tau_{\text{geom}}$, constant within an experiment and varied between experiments. For τ_{clock}= 1 and τ_{geom} = 2, these protocols are equivalent,

but this particular case was not studied as it would imply a very high density of pulses.

We suspect that the confusion may stem from the oversimplified notation used in Fig. 1A. To disambiguate, we modified labels: $\tau_{\rm geom} \longrightarrow \tau \sim {\rm Geom}(1/\tau_{\rm geom})$, $\tau_{\rm geom} + \tau_{\rm gap} \longrightarrow \tau \sim \tau_{\rm gap} + {\rm Geom}(1/\tau_{\rm geom})$.

> **5.** The work performs experiments with the plus duration 100 ms. Different plus duration may encode different mutual information. Besides, the study used one-minute resolution. Could the authors discuss more about the possible effects when changing plus duration and one-minute resolution?

Response: The analysis by Dessauges *et al.* [*Mol. Syst. Biol.*, 2022, DOI: 10.15252/msb.202110670], presented in Fig. 2A therein, indicates that for sub-second light pulses, the ERK response depends on the total light dose = pulse duration × light intensity. An increase in the light dose above some threshold slightly extends the duration of the ERK peak and may also increase the fraction of responding cells. We expect that a longer ERK peak would extend the refractory period and thus decrease the information transmission rate in individual cells.

Based on results showed in Fig. 1E and 1F, we expect that sub-minute temporal resolution may yield increased estimates of the lower bound of the information transmission rate. Using finer temporal discretization would substantially increase the entropy of the input, but in the reconstruction-based approach it would also result in more classification errors, therefore both $H(S)$ and $H(S|R)$ would be increased. Even for one-minute resolution, a substantial part of information loss is due to inaccurate detections (see Fig. 2D and 2F). We added this information to Discussion.

> **6.** In the Discussion, the author discussed the information transmission rate in other biological systems, for example, neurons, *E. coli* chemotactic system. Could the authors compare the information transmission rate between ERK pathway and these systems and then discuss the possible reason to cause such difference?

Response: The concepts of entropy, information, and information transmission rate do not exist without reference to a specific quantity of interest. Our selection of biological systems and associated estimates of the information transmission rate demonstrates that the same theoretical approach was proposed to characterize different biological systems and emphasizes that the rates may vary widely. The maximum bitrate depends on biophysics of a specific information channel. In the case of neurons, where the bitrates are highest, information is transmitted with the help of fast electro-chemical signals; in the case of *E. coli* and MAPK, information is transmitted by means of posttranslational proteins modifications, thus expected bitrates are lower. The bitrate observed in the *E. coli* chemotactic system can be higher than that expected for the MAPK channel, because bacteria is much smaller than an eukaryotic cell. Some

other signaling pathways, such as that of NF-κB or p53, require protein synthesis and degradation, which is a process slower than post-translational modifications, and so for these pathways even lower bitrates are expected.

> **Minor comments**
>
> 1. The description of the interval encoding protocol with a minimal gap in lines 82–85, "*...the time intervals were also drawn from a geometric distribution (with the mean interpulse interval τ_{geom}), but the intervals shorter than a minimal interval τ_{gap} were excluded...*", could be misleading. According to such a description, it's unclear how the expectation value of time interval equals $\tau_{geom} + \tau_{gap}$, as shown in Fig. 1A and in the method section.

Response: Due to the memoryless property of the geometric distribution, this procedure is equivalent to drawing τ from the geometric distribution with mean τ_{geom} and adding a fixed τ_{gap}, which implies that expected value of the inter-pulse interval is $\tau_{gap} + \tau_{geom}$. Because of this equivalence and because we find the latter explanation (with adding τ_{gap}) more straightforward, we rephrased the text about generating random pulses according to protocols from the third family (interval encoding with a gap).

> 2. The "*gapless protocol*" in lines 450–451 is confusing. Which one of the three types of pulsatile stimulation protocols does it indicate?

Response: To disambiguate, we changed: '*For the "gapless" protocol [...]*' ⟶ '*For the interval encoding protocol (nominally without a minimal gap) [...]*'.

> 3. On Page 22, "*For the "gapless" protocol, τ_{geom} was varied whereas τ_{gap} was set to 2 min (to avoid ambiguities in signal reconstruction). For $\tau_{geom} > 3.15$ min, $h_{interval}(S; \tau_{geom}, \tau_{gap})$ is a decreasing function of τ_{geom}...*". The paragraph is hard to understand and needs to be rewritten. For example, it's confusing that a "*gapless*" protocol has a $\tau_{gap} = 2$ min; it is unclear what is the purpose of varying τ_{geom}; how is the "*3.15 min*" determined?

Response: We have changed: '*[...] whereas τ_{gap} was set to 2 min (to avoid ambiguities in signal reconstruction)*' ⟶ '*[...] and a technical τ_{gap} of 2 min was introduced in experiments to avoid ambiguities in signal reconstruction*'. The 3.15 min was determined by differentiating Eq. (6) with respect to τ_{geom} and observing that for $\tau_{gap} = 2$ the function $h_{interval}(\tau_{geom})$ has a unique maximum in $\tau_{geom} \approx 3.15$. Nevertheless, the information seems unnecessary, and we removed it from the revised manuscript.

> **4.** In Fig. 1A, the way to plot time duration 5,6,21,3,18,... can be misleading. It would be more clear if a curly bracket is placed above these time durations or if these numbers are rewritten in a monotonically increasing way, for example 0, 5 min, 10 min, 15 min, 20 min, ...

Response: In Fig. 1A caption we added that: 'Small numbers in bold typeface denote intervals between pulse slots (in binary encoding) and inter-pulse intervals (in both interval encodings).'

> **5.** The legend for small gray bars in Fig. 1D is missing.

Response: We added the missing legend. The small gray bars denote false pulse detections.

> **6.** The y axis label "*Information transmission rate*" in Fig. 2 B, D, and F is confusing. The information transmission rate is the transmitted rate, that is, the black part of a bar, not the whole bar.

Response: We changed the the axis label to 'Lost and transmitted information rates'. Additionally, in Fig. 2B, 2D, and 2F captions we added: 'In each column, total height of the stacked bars is equal to the input entropy rate.'

Response to the Report from Reviewer 2

> In this paper, the authors measure a rate of information transfer through the Ras–ERK signaling pathway in fibroblast cells. This was done using an optogenetic stimulation system and ERK nuclear translocation reporter for measuring cell responses. The authors delivered three stimulus protocols in which signal variations were encoded differently (presence or absence of signal at each time; timing of signals; timing of signals with a minimum duration between consecutive signals). The mutual information between the stimulus trajectory and cell response trajectory was estimated using a classifier that was trained to detect when signals were delivered, and a mutual information rate was calculated by dividing by the total trajectory time. The authors estimate an information rate of 4–6 bits/hr under these protocols.
>
> My main concern is about the motivation and interpretation of these results. What is the biological meaning of this information rate? What makes it "*the*" information rate (line 271)?

Response: The information rate estimated in our study imposes a lower limit on the MAPK channel capacity. As the MAPK pathway coordinates multiple cellular functions, the transmitted information can be used for various purposes. Similarly, a neuron of the fly visual system transmits information about a stimulus at rates of up to 90 bit/s (Ref. 14 = Strong *et al.*, 1998, *Phys. Rev. Lett.*, DOI: 10.1103/PhysRevLett.80.197), and this information may be utilized in different ways. As we understand the Reviewer, the question is how a cell can utilize 6 bit/h of information transmitted via the MAPK channel. We think that the most information-consuming process coordinated by the MAPK pathway (mostly at the level of RAS) is cell motion. We intentionally estimated information using the reconstruction-based approach (please see also the response to Reviewer 1's major point 1), because the reconstruction allows us to observe that when a light pulse is at least 20 min after the preceeding one, it can be detected with 1-min resolution with at least 70% confidence (see Fig. 1G). This implies that majority of cells may respond in a coordinated manner to pulsatile stimulation with inter-pulse intervals not shorter than 20 min. Such synchronous responses to the stimuli may enable long-distance waves of ERK activity that assist wound healing, inducing collective cell migration (see Refs. 17, 18 and Discussion).

> The cell responses and thus the information rates likely depend on strength of optical illumination. How did the authors choose the illumination strength? How do the measured responses with this illumination strength compare to responses to physiological levels of chemical inputs? And thus, how does the measured information rate compare to what might be expected in a biological context?

Response: Typically, ERK produces pulses of the same amplitude, see, e.g., Albeck *et al.* [*Mol. Cell,* 2013, DOI: 10.1016/j.molcel.2012.11.002]). This is because ERK works as an amplifier of external stimuli and typically produces an all-or-nothing response. In Dessauges *et al.* [*Mol. Syst. Biol.*, 2022, DOI: 10.15252/msb.202110670] we found that by modulating the light intensity and duration, it is possible to produce intermediate responses of lower amplitude. However, this also increases response heterogeneity, due to the fact that some cells respond and others do not. To evaluate the maximal bitrate of transmission, we needed to be in a condition, in which the light pulse could reliably generate an ERK pulse. Thereby, we decided for a pulse of light short but intense enough to reliably generate an ERK pulse at maximal amplitude in all the cells of the field of view. A pulse of opto-FGFR stimulation at a chosen light dose leads to similar-amplitude ERK activation as the one observed during EGF tonic stimulation in range of physiological concentrations (1 ng/ml – 100 mg/ml). The peak of ERK activity in response to tonic EGF is observed in between 5 and 10 min after stimulation, similarly to the ERK peak observed in response to a light pulse. The post-peak trajectory observed in response to tonic stimulation is different. Of note, the type of cells used in our experiments is sensitive to flow (required for pulsatile stimulation with growth factors) and this is why we turned to optical stimulation.

> It is also hard for me to assess whether these protocols are close to anything cells would see in natural contexts, and thus to assess whether the measured information rates mean anything to the cell.

Response: We think (see revised Discussion) that the interval encoding protocol is a biologically realistic protocol. This protocol simply implies that the propensity for an input pulse stimulation is constant in time, equal to the inverse of the mean inter-pulse interval. This can be expected when stimulation comes from single neighboring cells (for example apoptotic cells as in Gagliardi *et al.* [2022, *Dev. Cell*, DOI: 10.1016/j.devcel.2021.05.007]). In the case of ERK activity waves one can expect that the first "layer" of cells filters out pulses that occur within the refractory time, and thus the farther "layers" of cells will be subjected to stimulation that resembles the interval encoding with a minimal gap.

> Several scenarios are mentioned in the Discussion where Ras–ERK signaling is used in higher organisms, but not all of those involve ERK nuclear translocation. It seems like the measured information rate is irrelevant to those processes. Maybe this work is relevant to synthetic biology? One might want to use the Ras–ERK pathway to send signals through a human-made biological device?

Response: In Discussion we refer to known processes involving signaling cascades, or neuronal signaling, for which the information rate was directly estimated, or can be estimated from the observed behavior. The examples with *E. coli* and Dicty were chosen to show that relatively high information rates are needed to govern cell motility in response to varied stimuli. We see, based on our unpublished data, that light can be used to direct cell motion (see movie "Realtime control of cell migration using an optogenetic FGFR receptor" at https://www.pertzlab.net/movies), which can be used for synthetic biology.

> **Other**:
> This paper and others before it lump together responses of different cells. But this is known to reduce the measured information transfer because cell-to-cell differences in responses are treated as noise, even if single-cell responses are very reproducible (*e.g.* as in the data of Toettcher *et al*, ref 22). The authors should comment on the difference between single cell and population-lumped measurements and what it means for how their results are interpreted.

Response: Yes, the Referee is fully right that by lumping all cell responses, a part of information is lost. We agree (and see in our data) that cell-to-cell differences interfere with pulse reconstruction. For example, we observe that there are faster and slower cells, and this causes that our predictor detects

pulses with 1 min delay, or 1 min in advance. We discuss this in the revised Discussion, referring also to Selimkhanov *et al.* [*Science*, 2014, DOI: 10.1126/science.1254933], where this effect was studied also in the context of information transmission (about a steady input).

> Related, is there communication among cells in this experiment, as it sounds like around line 289 in the Discussion?

Response: We cannot see communication between cells, just because all cells are equally illuminated. When, however, cells are stimulated at a given spot only, for example due to an apoptotic event, one may observe propagation of an ERK activity wave outside of this spot. This clearly indicates for cell-to-cell communication and propagation of information. See Gagliardi *et al.* [*bioRχiv*, 2022, DOI: 10.1101/2022.07.12.499734 (Fig. 4A). We include this information in Discussion.

> How was the 8-minute window size chosen for the classifier? Does this have biological significance?

Response: As showed in Fig. 3D and 3E, the 8-min window suffices to collect nearly all available information which is necessary to reconstruct an input pulse. From the biological perspective it means that a cell *does not need* to collect information (at the ERK level, or rather the ERK reporter level) for longer than 8 min to make the decision. Fig. 3D and 3E indicate also that nearly complete information is available 5 min after the input pulse. As the information is relayed through the kinase cascade, one could expect that upstream of ERK (for example, at the level of RAS) the information is available earlier and a shorter time window is sufficient.

> In the introduction and conclusion, the authors estimate an information rate of 1 bit per minute during Dicty chemotaxis. This estimation procedure only works if the cells always switch direction with the chemotactic gradient when it oscillates at 0.02 Hz. If there is any variability (incorrect decisions), the information rate must be lower.

Response: Yes, the Referee is fully right, our conversion implicitly assumed that all decisions are correct. We modified text in Introduction to: 'This suggests that the rate of information transmission through the navigation system is up to 1 bit per minute, and the limit is achieved if all cell decisions are correct.' In Discussion we wrote: 'This suggests the information processing rate of about 1 bit per minute, or less if some cell switch-or-not decisions are incorrect.' □
